# “Mobilizing our leaders”: A multi-country qualitative study to increase the representation of women in global health leadership

**DOI:** 10.1371/journal.pgph.0000646

**Published:** 2023-01-30

**Authors:** Claudia T. Riche, Lindsey K. Reif, Natalie T. Nguyen, G. Rinu Alakiu, Grace Seo, Jyoti S. Mathad, Margaret L. McNairy, Alexandra A. Cordeiro, Aarti Kinikar, Kathleen F. Walsh, Marie Marcelle Deschamps, Sandy Nerette, Smita Nimkar, Neema Kayange, Hyasinta Jaka, Halima M. Mwaisungu, Domenica Morona, Thandiwe Yvonne Peter, Nishi Suryavanshi, Daniel W. Fitzgerald, Jennifer A. Downs, Adolfine Hokororo

**Affiliations:** 1 The Haitian Group for the Study of Kaposi’s Sarcoma and Opportunistic Infections (GHESKIO), Port-au-Prince, Haiti; 2 Department of Medicine, Center for Global Health, Weill Cornell Medicine, New York, New York, United States of America; 3 Department of Medicine, Division of General Internal Medicine, Weill Cornell Medicine, New York, New York, United States of America; 4 BJ Government Medical College and Sassoon Hospital, Pune, India; 5 BJ Government Medical College–Johns Hopkins University Clinical Trials Unit, Pune, India; 6 Department of Pediatrics, Bugando Medical Centre and Catholic University of Health and Allied Sciences, Mwanza, Tanzania; 7 Department of Medicine, Bugando Medical Centre and Catholic University of Health and Allied Sciences, Mwanza, Tanzania; 8 Department of Internal Medicine, Mwanza College of Health and Allied Sciences, Mwanza, Tanzania; 9 Department of Medical Ethics, Catholic University of Health and Allied Sciences, Mwanza, Tanzania; 10 School of Public Health, Catholic University of Health and Allied Sciences, Mwanza, Tanzania; 11 Department of Administration, Catholic University of Health and Allied Sciences, Mwanza, Tanzania; Université de Montréal École de Santé Publique: Universite de Montreal Ecole de Sante Publique, CANADA

## Abstract

**Introduction:** Women play an essential role in health care delivery, and it is vital that they have equal representation in health leadership for equity, innovation, and the strengthening of health systems globally. Yet women remain vastly underrepresented in global health leadership positions, providing a clear example of the deeply rooted power imbalances that are central to the calls to decolonize global health. We conducted a multi-country study in Haiti, Tanzania, India, and the USA to examine gender-based challenges to career advancement for women in the global health workforce. Quantitative data on the type and prevalence of gender-based challenges has been previously reported. In this study, we analyze qualitative data collected through focus group discussions and in-depth interviews to understand women’s experiences of gender-based obstacles to career advancement, their perceptions of underlying drivers, and perspectives on effective solutions. Guided by an adaptation of the Social Action Theory, we conducted focus group discussions and in-depth interviews with women at 4 major academic centers for clinical care and research in Haiti, India, Tanzania, and the United States. In total, 85 women participated in focus groups and 15 also participated in in-depth interviews. Discussions and interviews were conducted in the local language, by an experienced local facilitator unaffiliated with the participating institution, between 2017 and 2018. Discussions were recorded, transcribed, and translated. Data were analyzed by interpretive phenomenological methods for emergent themes. Three transcendent themes on gender-based challenges were identified: 1) cultural power imbalance, referring to the prevailing norms and engrained assumptions that women are less capable than men and that women’s primary responsibility should be to their families; 2) institutional power imbalance, referring to the systematic gender bias upheld by existing leadership and power structures, and ranging from exclusion from career development opportunities to sexual harassment and assault; and 3) restricted agency, referring to women’s limited ability to change their circumstances because of unequal cultural and institutional structures. Participants also described local, actionable solutions to address these barriers. These included: 1) formal reporting systems for sexual harassment and assault; 2) peer support and mentorship; and 3) accessible leadership training and mandatory gender equity training. Participants proposed feasible strategies to address gender-based challenges that could improve women’s retention in health careers and foster their rise to leadership. Increasing the representation of women in global health leadership positions responds directly to efforts to decolonize global health and is integral to strengthening health systems and improving health outcomes for women and children worldwide.

## Introduction

There remains a lack of gender parity in global health leadership across governmental, non-governmental and academic institutions worldwide with the widest disparities exhibited in low- and middle-income countries (LMICs) [1,2]. Across 200 global health organizations, only 30% of leadership positions are held by women and only 1 in 6 of these (5% overall) is held by a woman from an LMIC [3]. In the World Health Assembly, the world’s preeminent health policy-setting body, less than a quarter of Member State heads of delegation are held by women [4]. In the response to the COVID-19 pandemic, which uniquely impacted women’s health and well-being, 85% of 115 task forces convened globally were comprised of more men than women [5]. This data underscores the stark reality that the perspectives of men from high-income countries remain the leading voices in global health [3,6]. Uprooting these asymmetric norms of power and male privilege is essential if we are to respond meaningfully to the growing calls to decolonize global health–that is, to recognize and dismantle the oppressive, colonialist legacy embedded in the patriarchal structure of global health [7–9].

Gender imbalance in science and health limits diversity of scientific inquiry which subsequently constrains research findings to apply best to the populations studied, marginalizing the health of populations not included [10]. Critically, an analysis of over 11.5 million scientific papers published between 2008 and 2015 demonstrated that authors’ gender directly influences whether they report on sex-related differences, with female first and last authored-papers being most likely to report results by sex [10,11]. The critical inclusion of women in health research and leadership has been the subject of Policy Action Papers by the WHO and has spawned the establishment of the UN Women Generation Equality [12,13]. Gender inequity in health care has a deep-rooted history. The first woman to graduate medical school in Tanzania was in 1969 and across Africa today, just 28% of physicians are female [14]. In In the US, it wasn’t until 1993 that the National Institutes of Health in the United States required the inclusion of women in government-funded research [15], by which time the scientific world was already decades behind in understanding women’s health compared to men’s health across a variety of disciplines. At a minimum, sex-disaggregated data is indispensable for understanding sex differences such as appropriate diagnoses, medication dosing, and intervention effects in clinical and public health data [16–18]. Despite the growing global consensus that more women in global health leadership are deeply needed, little data exists on the lived experiences of the very women who would rise to leadership–the women trainees and faculty members in LMICs [12].

This study responds to the urgent need to amplify the voices of women in global health, with an emphasis on those from LMICs. Prior work has quantified the challenges preventing women’s promotion to leadership positions in global health, with more than three-fourths of women in the field reporting challenges with work-life balance, and more than half reporting facing gender bias and discrimination, and sexual assault [19,20]. Only limited qualitative research which explores the spectrum and severity of barriers has been conducted. To address this gap, we conducted focus group discussions and in-depth interviews among women working in global health in four countries (Haiti, India, Tanzania, and the United States). We sought to examine women’s experiences of gender-based obstacles to career advancement, their perceptions of the underlying drivers of these obstacles, and their perspectives on effective strategies for the future.

## Methods

This research was carried out according to the Standards for Reporting Qualitative Research (SRQR) and we have adhered to these guidelines and the 21-item checklist for reporting the study’s results (S1 File and S2 File) [21].

### Study setting and population

This study was conducted at four major academic centers for clinical care and research with national and international funding in four countries. The Haitian Group for the Study of Kaposi’s Sarcoma and Opportunistic Infections (GHESKIO) in Port-au-Prince, Haiti is the largest HIV/AIDS clinic in the region providing care to over 600,000 patients annually. Byramjee Jeejeebhoy Government Medical College (BJGMC) in Pune, India is a major tertiary care facility providing care for over 60,000 inpatients and over 525,000 outpatients annually. Bugando Medical Center (BMC) at the Catholic University of Health and Allied Sciences (CUHAS) in Mwanza, Tanzania is one of four major medical centers in the country and serves a population of 15 million patients. Lastly, Weill Cornell Medicine (WCM) in New York, USA has partnered with each of these sites to collaborate on the conduct of global health research, training, and clinical service for decades. Focus group discussions and in-depth interviews were conducted at off-campus locations in the local language (i.e., Haitian Creole, Hindi, Kiswahili, and English) by experienced local facilitators who were unaffiliated with the participating institution.

### Data collection

Female health trainees and faculty from each institution were recruited to participate in a study exploring gender-based challenges to career advancement for women in global health. All study participants completed a confidential quantitative survey to determine the type and prevalence of gender-based challenges experienced in the global health workforce, of which the results have been previously reported [20]. Local study coordinators advertised the study at each institution through flyers, emails to institutional mailing lists, and announcements in classrooms/shared spaces at each site. Interested participants were invited to contact the study coordinators in person or by email or WhatsApp. Participants were also encouraged to invite other potential respondents by snowball sampling [22]. Among a subset of participants who expressed interest in further participation, study coordinators organized focus groups discussions and in-depth interviews. The goal of this qualitative data collection was to understand their experiences of gender-based violence, their perceptions of the underlying drivers, and identify solutions that would address these gender-based challenges. We began with focus group discussions to provide a forum in which women could share experiences and secondarily used in-depth interviews to gather additional data if participants felt uncomfortable sharing in a group setting. Focus group discussions consisted of approximately 5–10 participants, lasted 45–60 minutes, and were stratified by training level (student, post-graduate trainee, or faculty) to encourage sharing of experiences among peers. Individuals who indicated interest participated in additional confidential in-depth interviews following the group discussions, which lasted 30–45 minutes each. Discussions were digitally recorded, transcribed into the local language, and translated into English by professional translation services.

The content of the structured questions used to guide the focus group discussions and in-depth interviews was based on a thematic analysis of data from three large public symposia focused on Women in Global Health Research that occurred between 2015 and 2017. These symposia were organized by study team members from each of the institutions that participated in this study and were attended by over 180 female global health trainees and professionals from seven countries, including LMICs (womenglobalhealth.com). The symposia included panel discussions and small group sessions during which attendees discussed challenges that prevent women from pursuing global health careers. Videos of all talks and panel discussions were recorded, with detailed minutes of the group discussions. In multiple study team meetings, our leadership team (CR, LR, JM, NS, JD, AH), which included women from all four study sites, analyzed data from these symposia. Through group consensus, the team identified three major themes that guided the development of the focus group and interview questions used in this study: gender discrimination, work-life balance, and sexual harassment or assault. Questions were reviewed with the study team in each country to ensure they were appropriate. Qualitative interviewers received training in strategies to avoid and mitigate bias during data collection.

### Data analysis

We performed a thematic analysis using interpretive phenomenological analysis to explore participants’ perspectives on their lived experiences as women during their training and work [23–25]. English transcripts were imported into Nvivo Version 10 (QSR International, Doncaster, Australia), which was used to organize study data and codes. Data were initially analyzed and independently coded by two authors, NN and RA, to identify and agree upon emerging themes. In vivo coding was used for themes that emerged during coding. Codes were compared every two weeks during the coding for refinement and clarification by study team members, CR, LR JD, and AH, and differences were resolved via group process. Through repeated examination, an iterative review of the themes, and grouping of codes, we identified prevailing themes that represent women’s perspectives on gender-based challenges experienced during their training and work and associated solutions to these challenges. We continually monitored for data saturation and our analyses indicated data saturation was achieved. Participant quotations illustrating these themes were selected by group process and are presented here.

### Theoretical framework

We used the Social Action Theory (SAT) conceptual framework to organize qualitative data related to increasing women in global health leadership, as previously described [20]. This framework incorporates experiences at the contextual, institutional, and individual levels to describe cumulative effects on, as well as to identify strategies for, interventions and outcomes [26,27]. We adapted this model to include participants’ environments, such as local cultural norms, discourse around gender equity, and institutional policies regarding gender discrimination and harassment. Additional adaptations incorporated social interactions such as the availability for peer support and the role of mentors. We used the themes identified from the analysis of our symposia to map our qualitative data thematically to the framework. We additionally adapted components of the framework to include other important influences on social action, such as environment, institution, individual, and social/peer support.

### Ethical considerations

Institutional Review Boards at GHESKIO, BJGMC, BMC/CUHAS, the Tanzanian National Institute for Medical Research, and WCM approved this study. Written informed consent was obtained from all participants of the focus group discussions and in-depth interviews. Participants were asked about their experiences during all stages of their training but did not provide identifying information including institutions, locations, or dates of training to protect participant confidentiality. Any identifying information was deleted after data collection was complete. The survey did not differentiate experiences at previous and current institutions. Qualitative data was de-identified during transcription and translation and stored on password protected computers. After analysis, recordings of focus groups and in-depth interviews were destroyed and three years after date of publication all written data will be destroyed. De-identified data from all four sites were compiled after data collection; individual sites were not identified in any analyses to protect participants’ confidentiality.

## Results

Between February 2017 and January 2018, 653 women were invited to participate in the larger study and 346 (53%) completed the survey (Table 1). Among these, 85 (25%) were willing to participate in additional focus group discussions and of those, 15 (18%) also agreed to additional in-depth interviews. The number of participants per country was limited and to ensure confidentiality, characteristics of participants of focus group discussions and in-depth interviews are not provided. A total of 17 focus group discussions were conducted, of which 4 were among students, 4 were among post-graduate trainees, and 9 were among faculty or health professionals.

**Table 1:** Respondent characteristics*.

	Total (N = 346)
**Career Stage (N, %)**	
Students	120 (35%)
Postgraduates	90 (26%)
Faculty/Professional	136 (39%)
**Age in years (N, %)**	
Median (IQR)	29 (24–35)
<25	94 (27%)
25–40	210 (61%)
41+	42 (12%)
**Marital Status (N, %)**	
Married	128 (37%)
Unmarried	218 (63%)

*Represents participants in the larger study [20]. Among these, N = 85 participated in focus group discussions, 15 of whom also participated in in-depth interviews. More detailed characteristics not provided to protect anonymity of respondents.

Three primary themes related to gender-based barriers emerged: cultural power imbalance, institutional power imbalance, and restricted agency. Two cross-cutting sub-themes were also identified that were observed in several of these categories: motivation to seek leadership roles and balancing maternal and family responsibilities with career advancement.

### Cultural power imbalance

Cultural power imbalance refers to engrained ideas, reported from all countries, that women are less capable than men, and that the normative roles of women are to maintain their households and raise children. These core, pervasive beliefs undermined women’s motivation to even a seek leadership role. They described having their inferiority to men impressed upon them since childhood. Even a senior faculty member explained:

*“There is a lot of discrimination*. *[Society] is saying that a man is born to be a leader and a woman is born to be a slave under a man*, *so that discourages women a lot—that they are created to be under men…A man must be above you…most of the time in anything that you do*, *as long as a man is there then you will not lead*.*”* (faculty member)

Women at all stages of their careers stated that their career paths had been arduous, requiring them to fight against pervasive cultural norms and continually prove themselves. Participants expressed their struggle to gain respect juxtaposed with the respect perfunctorily offered to men: “*When women doctors order men*, *they do not obey*. *But if it was a man who had ordered them*, *they would have obeyed…we women have to shout*” (faculty member). Women frequently shared instances in which they or their colleagues were passed over for leadership positions or promotions in favor of men with less experience. Many participants explained that they had been discouraged from male-dominated specialties or penalized for childbearing. When asked about instances of gender discrimination, a surgical trainee stated that:

*“Our society perceived [women staying at home] as a normal thing*. *If you work*, *it will be like you are doing a supplementary thing*. *If they see you [at work] they will ask*, *‘What have you come to do*? *You*, *a woman*, *are going to operate on somebody*?*’”* (surgical trainee)

Further, these prevailing cultural beliefs led women to feel that *“we are devalued by the society*, *and this belief has been there a long time”* and at times led to decisions not to pursue leadership roles in a society that *“does not believe women can be given leadership*” (faculty member). A trainee observed that even when men and women actually did achieve *“similar [leadership] positions… the women are not as respected”* (post-graduate trainee). Another trainee described her perception that these biased cultural norms extend to leaders of institutions who commonly think, *“women*, *women*, *we don’t trust women”* (post-graduate trainee).

This experienced cultural power imbalance was further exacerbated for pregnant women. Multiple respondents stated that women who become pregnant are perceived to be less serious about school and work. Both students and post-graduate trainees described women being chastised by their superiors, sometimes in front of others, for becoming pregnant during training. One trainee explained that, after returning from maternity leave, *“[The seniors] will ask you more questions just to teach others that they should not [become pregnant] like you”* (post-graduate trainee). Another trainee described pervasive gender bias by stating, *“by the way they treated women who became pregnant*, *we can feel it”* (post-graduate trainee). Repeatedly observing and experiencing that pregnancy was not compatible with training for a career in medicine discouraged women from considering career advancement or leadership.

### Institutional power imbalance

A second major theme that emerged in both focus group discussions and in-depth interviews was the existence of institutional policies and practices that enable gender discrimination and harassment. Women described occasions on which superiors used their institutional power to threaten or block them from advancing in their careers or excluded them from training opportunities altogether. An orthopedic trainee reported that her superiors *“failed to give the orthopedics examination”* needed to advance her career, hoping that she would *“get tired and leave*.*”* They asked her, *“Why can’t you go and study pediatrics*?*”* Several women recounted being forced to cover a male colleague’s patients so that he could attend a training seminar, while not being given opportunities to receive training themselves. Again, the pervasive assumption of male superiority and women’s perpetual struggle to get equal treatment sapped motivation to fight these inequities and pursue leadership.

Institutional power imbalance also manifested the cross-cutting theme of balancing maternal and family responsibilities with career advancement. Because trainees are expected to adhere to a rigid training and examination schedule, a maternity leave of several months is currently not possible in many programs. For example, one medical resident was given one week of maternity leave during her residency training and when she asked for additional time was told to “*choose school or postpone [her studies] a year*.” To avoid this unanticipated year-long delay, she returned to her training program before she had recovered from her delivery and described working through physical pain. A faculty member described getting a breast abscess *“because of not feeding the baby on time”* when she was not permitted a break during her shift as an intern. Of note, no participant reported having discussed her plans for childbearing with a mentor, or seeking guidance on career-family balance from a mentor, while in her training.

Similarly, participants who had recently returned from maternity leave reported that their careers had been stalled because of their childbearing. A post-graduate trainee stated that *“ultimately the accolades go to men because they just did not physically have to bear children and miss out on the opportunities*.*”* Another post graduate trainee described maternity leave hindering a woman’s chance for promotion, stating that, *“I hear employers do not like those maternity leaves*. *If there is [a chance] to choose leaders and you have gone on maternity leave*, *you cannot be given [the promotion]*, *know that leadership has passed you*.*”*

Men’s power in some institutions further extended to their ability to coerce women into performing sexual acts in order to avoid negative consequences such as unwanted transfers, failing courses, or lack of promotion. One faculty member reported that the head of her institution approached her and threatened to *“arrange [to send] her to the village*,*”* an undesirable site to work, if she did not agree to his sexual advances. Another faculty member stated: *“Your teacher will disturb you because…when you refuse his advances…you fail his course*, *so he uses that as an entry point*.*”* A medical student crystallized the challenge of professors’ unchecked authority when she stated, *“At this level*, *your future is in the hands of your teacher*, *the one harassing you*.*”*

### Restricted agency

These cultural gender biases and institutional power imbalances fueled a sense of restricted agency among many participants. Common sentiments in discussions included feelings of futility and resignation in response to prevailing cultural attitudes that normalize gender discrimination, constrain balance between family responsibilities and career opportunities, and tolerate sexual harassment in the workplace. These culminated in the disheartened assumption that since women are unlikely to overcome these challenges, many are not motivated to seek them in the first place. A medical student expressed many women’s resignation to accepting the default of male leadership:

“The words of many people make us fear that they would say ‘This woman isn’t able.’ Because many people have seen that leaders are men…Therefore we remain back, leaving men alone to lead us.”

Participants also described that the combination of unsupportive family policies at work, plus their disproportionate share of family responsibilities compared to their male partners, discouraged them from even trying to pursue leadership positions. One medical student remarked, “*[she] cannot do many things at once…therefore women back up from getting into leadership positions*.*”* Others perceived that the emotional weight of balancing work, studies, home, and family limited women’s ability to be fully present for work and career activities. One faculty member described: *“You find women’s performance is dropping because they can’t concentrate in studies and work… the child has a fever; the child is not fed*.*”*

Futility was expressed most prominently in instances of sexual harassment or assault in the workplace, which were well-known and frequently described. When asked what women do in response to sexual advances from superiors, participants repeatedly noted the absence of reporting or support systems at their institutions. Without reporting mechanisms, “*most women remain silent*” (post-graduate trainee) and resort to seeking support and comfort from peers: *“There is no special authority where we can report these problems…we remain encouraging each other alone”* (faculty member). Another trainee echoed this sentiment, stating that “*there is not any system…you just look to your friend to talk about that heartache*” (post-graduate trainee).

Further, lack of formal reporting results in lack of consequences for perpetrators and women subsequently lose out on opportunities. One faculty member described that after rebuffing her supervisor’s demand for sex in exchange for his letter approving her request to transfer work location, she “*ultimately gave up trying to transfer*.” A faculty member described a tragic example of the potential consequences of sexual harassment when students have no recourse and no help:

“[She] loses concentration and psychologically she becomes affected…[it] may cause absenteeism, she doesn’t enter the classroom, she is afraid of meeting that teacher. Therefore, the absenteeism leads her to fail studies and finally leave the college altogether.”

### Proposed solutions to address barriers

Women’s practical suggestions for addressing these challenges can be summarized into three key interventions: a robust reporting system, peer support and mentorship, and gender equity and leadership training. Their suggestions are summarized in Table 2 and described in detail below.

**Table 2:** Women’s recommendations for interventions to address gender-based barriers.

Intervention	Recommendations	Representative Quotes
**Robust Reporting System for Sexual Harassment and Discrimination**	*Establish system* • Institute clear, accessible and consistent formal reporting systems. • Provide clear definitions of harassment, discrimination, and prohibited conduct in the workplace. • Provide clear steps for appropriate disciplinary measures, including steps for human resources or other authoritative body to enact, for individuals who violate harassment policies. *Ensure functionality* • Provide a safe, reliable location and system in which students, faculty, staff and trainees can report observations of or sexual harassment experiences. • Ensure confidentiality of those who report sexual harassment. • Implement quarterly climate surveys and annual reports to quantify the: a) prevalence of harassment; b) types of violations reported; c) number of reports under investigation; d) descriptions of disciplinary actions. • Offer referrals to individual counselling services with trained counselors to assist victims of sexual harassment.	*“There must be more than one person with the authorized power on top of us*.*”* *“Laws [are needed] to protect women–and the laws must work and not be laws only written in books*. *If a person goes to claim any problem*, *it should be taken seriously and not a women’s issue*.*”* *“In an institution*, *there must be a code of conduct or specific laws*, *and [offenders] must be charged—expelled from work or [similar action] depending on the offense—this will help women*.*”*
**Peer Support and Mentorship**	Form and support peer groups • Establish peer group forums for students, staff, faculty and trainees to encourage solidarity with other women, and share experiences. • Provide protected time (e.g., twice monthly for one hour) for women’s peer group meetings during working hours. Promote female mentorship • Design mentoring programs for women to facilitate exchange of ideas, promote engagement in research, build skills, and improve promotion and retention. • Mentorship models can include: dyad model (one mentor/one mentee), group mentorship, peer mentoring to enhance feasibility. • Include career-family balance in mentorship discussions beginning early in women’s careers.	*“I agree with the issue of unity*, *in that unity we keep on educating each other…we can help on how to deal with [problems] in this way*.*”* *“This [organizing together] can give them strength*.*”* *“Make groups of women and speak to them about the importance to educate themselves and tell them about your own path if possible*.*”*
**Gender-Equity Education and Training**	• Provide gender-based leadership training for women at all levels (students, fellows, faculty, senior leaders). • Mandate training on the definition of sexual harassment and discrimination, including when and how to report it, for all members of academic institutions with support and mobilization from senior institutional leadership. • Develop training modules for how to intervene when witnessing sexual harassment and assault to normalize and facilitate stopping and preventing it.	*“This must begin with women because we are letting ourselves down…We should begin ourselves receiving the trainings*.*”* *“We women are always considered inferior…[so] we must learn to be empowered and encourage one another*.*”* *“There must be mobilization from our leaders*, *especially those who are women…at the end of the day*, *women are redeemers of the nation*.*”*

#### Robust reporting system

Participants reported that one of the prominent gaps enabling gender-based barriers to persist was the lack of formal reporting systems for sexual harassment and discrimination. “*There is no specific place I would be able to report…not to people who would be able to make a direct intervention*” (faculty member). For others, if a reporting system did exist, they felt that it was primarily symbolic and did not effectively hold perpetrators accountable–“*I think laws to ensure women’s rights exist but they are not enforced–there must be enforcement for the law to be effective*” (faculty member).

Instead of *“one person with the authorized power on top of us*” (medical student), women wanted a formal and functional reporting mechanism that would ensure anonymity and hold perpetrators accountable. Participants stated that the reporting system should be based on core qualities: anonymity, accountability, formal laws and procedures, managed by personnel unaffiliated with the institution, publicly accessible, and of highest integrity. One post-graduate trainee described the reverberating and sustainable impact a functional and reputable reporting system could have:

“*When [reporting] happens, the responsible are judged [and] others will stop [since] they know there are consequences*. *Therefore, if there is a strong organization that would support women, it may change the whole community*.”

#### Peer support and mentorship

Participants also suggested forming organized peer groups to provide support. While participants stated that they had friends or colleagues who support them through difficulties, they expressed a desire for the establishment of formal women’s groups to share perspectives, experiences and support. Women envisioned that these peer groups would enable women to advise one another and that “*in that unity we keep on educating each other*” (faculty member). One post-graduate trainee stated that:

“*There is power [in] speech. Make groups of women and speak to them about the importance [of educating] themselves and tell them about your own path if possible*. *How you managed to make it*.”

Mentorship from senior female role models was noted as particularly transformative. Participants emphasized senior women’s valuable role in supporting and encouraging younger women to combat cycles of undervaluing themselves or assuming leadership aspirations are futile. Enhancing the visibility of senior women provides practical examples for junior trainees that “*despite being a woman we can pass years of study without having to repeat exams and be among the best”* (medical student).

#### Gender equity training

Lastly, respondents expressed the necessity of educating the society at large about the value of women. They believed that cultural gender bias against women was taught from a young age and that changing this narrative through education is imperative for breaking systemic unequal gender norms. Women recognized that they themselves needed training, stating that *“we must empower women and [learn to] empower ourselves and encourage ourselves”* (medical student). Further, women asserted that there must be *“mobilization from our leaders*, *especially those who are women…*.*at the end of the day*, *women are redeemers of the nation*” (post-graduate trainee).

## Discussion

To our knowledge, this is the first multi-country study, conducted among women across low, middle-, and high-income countries, to qualitatively assess gender-based barriers women experience in attaining global health leadership positions. Across four economically and culturally diverse countries, women at academic centers reported strikingly similar gender-based barriers toward career advancement. Women’s career progression was consistently impeded by the imbalance of power, both culturally and institutionally, which translated to their restricted ability to effect change. Gender bias, unequal burden of family obligations, and sexual harassment have been documented as factors that limit women’s career progression worldwide [20]. Furthermore, gender stereotypes are compounded by race, ethnicity and socioeconomic position, all significantly impacting women’s rise to and performance in leadership roles [28]. This qualitative analysis of women’s experiences, and their perceptions of the underlying drivers of these challenges, also demonstrated remarkable overlap across disparate cultural and economic settings. Seeking out and amplifying some of the most marginalized voices–those of women from LMICs–provides a fundamental roadmap for urgently-needed change. If we are to address inequality in leadership positions in global health, we must start by heeding these voices.

Both cultural bias and institutionalized gender discrimination are pervasive globally, and multiple studies suggest that these forces impede women’s engagement, productivity and advancement to academic leadership positions [29–32]. Our findings are consistent, indicating that gender discrimination is systemic and hinders women’s career progression. A recent mixed-methods study of 227 PhD graduates in 17 sub-Saharan African countries found that women who got married or became mothers during PhD training had significantly fewer publications and took longer to complete PhD training compared to men. The opposite was observed among men—marriage and becoming a father during PhD training were associated with increased publication productivity and earlier PhD completion. Factors that were positively associated with earlier completion of doctoral training for women included: having a female supervisor, attending an institution with gender policies, and pursuing a PhD in a department where sexual harassment by faculty was perceived as uncommon [30]. Similar findings have been observed in academic institutions in high-income countries among women in science, technology, engineering, and medicine (STEM) [31–33], with common barriers also including unequal share of familial responsibilities, lack of family members’ understanding of work demands, perceived expectations of female faculty compared to males, and lack of female mentors [31,32]. Taken together, these studies and ours begin to quantify and vocalize the cultural and institutional factors that demotivate women to aspire and apply for leadership positions. To advance women into leadership roles, coordinated action at the levels of the individual, institution, and community must address these multi-level biases [20,34].

Sexual harassment or assault was experienced by 38% of women at the four academic institutions at which the quantitative portion of this study was conducted, demonstrating how pervasive this problem is globally [20]. Our qualitative data from these same women contributes to the limited qualitative data on this topic from LMICs, and provides context to the damage caused by harassment on women’s mental health, productivity, and, ultimately, career retention and progression [35,36]. Several studies from high-income countries, including a survey conducted by the US National Institutes of Health, have similarly found that women who experienced harassment were hesitant to report experiences out of fear that their careers would be penalized or because they perceived reporting systems as inaccessible or inept [31,32,37]. Implementing functional reporting systems to hold perpetrators accountable is a fundamental first step toward addressing the global crisis of sexual harassment [38,39]. Effective institutional systems implemented by conscientious institutional leaders are essential, particularly since 59 of the world’s 195 countries do not have laws prohibiting sexual harassment in the workplace and leave women in health care professions vulnerable [4]. Funding agencies should require that recipient institutions have written sexual harassment policies, formal procedures for violations, and regular obligatory training on discrimination and harassment for all trainees and employees.

Our data contributes to the extremely limited evidence-based research on interventions to address gender discrimination, harassment, and assault in academic institutions in LMICs [19]. Participants in our study suggested a package of four major approaches that should be implemented simultaneously to increase female retention, productivity, and advancement in health careers. Their solutions are practical, locally-driven, and relatively inexpensive (Table 2). First, formalized reporting systems must be designed and enforced by existing leadership in institutions. Given that a majority of institutions are currently led by men, which could present as a challenge in some countries, garnering their partnership to support these systems is essential. Second, peer mentorship groups can fill a mentorship gap that exists due to the dearth of female senior mentors. Peer groups can be informal, but institutional support to formalize them, providing space and time within the working day, increases accessibility and contributes to normalizing support for women in health care. Third, family-friendly institutional policies that simultaneously support women’s professional and personal roles will have high impact given that balancing career and family life is the most widespread and prevalent barrier women report [20]. For example, research-enabling grants can allow women to hire research personnel during maternity leave to prevent delays in productivity, and offering training both in-person and virtually increases the likelihood that it can be accessed by women who cannot travel [30,40]. Finally, it remains critical that gender equity training integrates decolonized approaches to global health that address histories of entrenched imperialism, racial and ethnic inequities, with the aim to empower individuals and bystanders to stand up for their women colleagues, and re-enforce support for these interventions.

Addressing gender inequity is fundamental to efforts to decolonize global health. It will contribute to restructuring an underlying system that was borne from a colonialist, patriarchal history that disenfranchises women. Increasing the number of women in LMICs in leadership in global health will also put women in positions of power, affording them opportunities to lead this restructuring in pursuit of a more equitable system. Research shows that female leaders make decisions which prioritize women’s health and well-being including access to education and immunization programs and addressing complex social and economic determinants of health that underpin poor women’s health outcomes [41,42].

A limitation of this study is that social desirability bias may have affected women’s honesty in sharing their perspectives. Women were asked to share about their own or their peers’ experiences, which may have lessened this bias and facilitated transparency. The candid discussion in many groups suggests that this strategy may have lessened this limitation. Secondly, participants were recruited through email invitation, flyers, and announcements, as well as by snowball sampling. Selection bias may have excluded women who feared repercussions of participating, who had experienced severe trauma they felt unable to share, or who felt that they had not faced gender-based challenges, or may have over-estimated the burden of challenges if the most vocal participants were more motivated to participate, though this limitation may be partially mitigated by consideration of the broader literature. Additionally, by recruiting from academic institutions we did not include the perspectives of women who had dropped out of their fields, though women who did participate described these occurrences among their colleagues.

Since we recruited from academic institutions, it is likely that our recruitment pool included women from higher socioeconomic backgrounds and who had better access to higher education than many other women in their countries. Thus, the study population should not be considered representative of the general population. Overall, we were able to include a large sample size for a qualitative study, and to reach data saturation, while including opinions from culturally and ethnically diverse individuals from four different countries. Further, factors specific to each country’s context as well as participants’ experiences within each country’s cultural setting (e.g. issues related to class, ethnicity, gender or sexual identity, socioeconomic status, etc.) did not emerge from the data as key findings. This may be due to minimal prompts from the interview guides on topics related to intersectionality and does not reflect the absence of the experience of the multiplying effect of intersectional issues in addition to gender.

### Future directions

Part of the solution to increasing female representation in health leadership requires that all voices be heard equally. However, there remains a paucity of research on male perspectives on gender disparities in health leadership. As men hold a majority of global health leadership roles, it is essential to engage them in conversation towards closing the gender gap in leadership. As such, future research should qualitatively explore male perspectives on gender-based differences in career progression and the role men can play in supporting equality in leadership. Likewise, other qualitative studies would facilitate understanding of gendered obstacles at different levels, including governmental, non-governmental, private, urban and rural health facilities. Furthermore, to assess the impact of interventions being implemented, we will seek participants’ follow-up testimonials and surveys to understand attitudinal shifts, experiences, and challenges as numbers of female leaders in the workplace increase. Lastly, future research on the intersectionality of gender with race, sexual identity and orientation, socioeconomic status, cultural background and other identities will provide a deeper understanding of the roles of these identities on career advancement and equity.

## Conclusions

Addressing gender inequity in global health leadership remains integral to decolonizing global health, and the courageous voices of women in this study offer guideposts for the way forward. Women’s equal representation in health leadership in LMICs, where a majority of the world’s most marginalized populations live, will contribute to dismantling systemic, ensconced gender biases. Further, gender parity in leadership will make meaningful impact on cementing women’s rights and will improve the health of women and children worldwide. As more women hold leadership positions, we can begin to reshape global health.

## Supporting information

S1 File Standards for Reporting Qualitative Research (SRQR). (PDF)

S2 File Inclusivity in global research. (DOCX)
